# Integrating nonstationary behaviors of typhoon and non-typhoon extreme rainfall events in East Asia

**DOI:** 10.1038/s41598-017-04629-1

**Published:** 2017-07-11

**Authors:** Chanyoung Son, Taesam Lee, Hyun-han Kwon

**Affiliations:** 1 Department of Civil Engineering, ERI, Gyeongsang National University, 501 Jinju-daero, Jinju, Gyeongsangnam-do, South Korea; 2 Water Resources Research Center, K-Water Institute 125, Yuseong-daero, Daejeon, South Korea; 3 Department of Civil Engineering, Chonbuk National University, Deokjin-dong 1ga, Deokjin-gu, Jeonju-si, Jeollabuk-do, South Korea

## Abstract

Extreme rainfall events in East Asia can be derived from the two subcomponents of tropical cyclones (TC) and non-TC based rainfall (mostly summer monsoons). Critical natural hazards including floods and landslides occur repeatedly due to the heavy rainfall associated with the two subcomponents, and disaster losses are increasing because global warming has caused changes in the extreme rainfall characteristics of two subcomponents. Subsequently, the frequency and intensity of extreme rainfall have reportedly become nonstationary. The majority of literature on nonstationary frequency analyses do not account for the different behaviors (stationarity or nonstationarity) of annual maximum rainfall (AMR) from the two subcomponents (*PM*_*TC*_ and *PM*_*NTC*_). To carry out a nonstationary frequency analysis considering the different behaviors of the *PM*_*TC*_ and *PM*_*NTC*_ series, this study proposes a novel approach of integrating the fitted *PM*_*TC*_ and *PM*_*NTC*_ series after modeling the nonstationarity of the *PM*_*TC*_ and *PM*_*NTC*_ series individually. The presented results conclude that the proposed approach provides more reliable estimates than existing nonstationary approaches by reflecting the different features of the *PM*_*TC*_ and *PM*_*NTC*_ series. We suggest that the proposed approach provides a reasonable design rainfall in constructing hydraulics to mitigate the different nonstationary effects of two TC and non-TC rainfall extremes.

## Introduction

Critical water-related natural hazards including floods and landslides occur repeatedly across this region partially due to the two subcomponents: tropical cyclone-induced rainfall (TC rainfall) and summer monsoon rainfall (non-TC rainfall)^1^. In addition, it has been reported that annual maximum rainfall (AMR) obtained from the two subcomponents in recent years exhibit nonstationarity due to climate change around the world^2, 3^. Kunkel, *et al*.^2^ reported that TC intensity and rainfall may increase in the United States. Knutson, *et al*.^3^ analyzed the changes in TC power dissipation in various tropical storm basins (e.g., Atlantic, Pacific) from seven global models, and all of the global models showed that the intensity of TCs generated in the western North Pacific (WNP) region affecting East Asia might increase. However, there was no mention about the variation of rainfall amount. Tu, *et al*.^4^ reported that strong TCs (category 4 and 5) have tended to occur more frequently in May since the year 2000.

Zhang, *et al*.^5^ reported that distinct decreases in rainy days were observed, but rainfall intensity increased over most parts of the Yangtze River Basin in China. Kim, *et al*.^6^ analyzed decadal variability in rainfall over South Korea from 1960 to 2010. They concluded that rainfall amounts tend to increase in summer seasons. In addition, summer monsoon rainfall over East Asia including central and northern China, the Korean Peninsula, and Japan is expected to increase due to climate change^7, 8^. Kripalani, *et al*.^7^ examined the future projection of summer monsoon rainfall under the radiative forcing of doubled CO_2_ scenarios simulated by the multi-model ensemble (MME; 22 coupled climate models) in the IPCC AR4 database, and the MME result revealed an increase ranging from 5 to 10% over the East Asian region. In particular, the increase is significant over South Korea, Japan, and northern China. Kusunoki and Arakawa^8^ investigated future changes in intensity of summer monsoon rainfall over Japan and the Korean peninsula and concluded an increase of rainfall intensity using future climate simulations (A1B emission scenario) by Couple Model Intercomparison Project 3 (CMIP 3) MME.

To mitigate the impact of extreme TC and non-TC rainfall events, flood control systems (e.g., hydraulic structures) have been installed with design rainfall and flood estimates. Hydraulic structures are generally designed based on the AMR series with the stationary assumption^9–11^. However, the assumption of stationarity in frequency analysis is questionable, and new frequency analysis methods that allow for nonstationarity in a given distribution parameters are required^12–15^. To treat nonstationarity of extreme events (e.g., rainfall and flood), several frequency analyses have been published in the literature, in which the parameters of a given distribution may vary over time^13, 16–28^. Regardless, the AMR events obtained from different subcomponents, TC and non-TC rainfall, extreme rainfall events from TC and non-TC have not been studied separately in the majority of literature available on nonstationary frequency analyses^9, 16, 18, 25, 29^. Recent studies related with the nonstationary frequency analysis for South Korea does not take those differences into account^29, 30^ because the difference of two weather systems (TC and non-TC extreme rainfall events) has not been further studied and the current model capability does not allow combining different systems.

In cases in which one of the two subcomponents contains an increasing trend whereas the other shows a decreasing trend, the traditional nonstationary frequency analysis could lead to an unrealistic representation of the design rainfalls (or floods). More specifically, the combined weather system might present stationarity or ambiguous nonstationarity by cancelling each other out in the trends. In addition, the AMR series for the rainfall frequency analysis are routinely constructed from the largest rainfall totals corresponding to different durations in each year so that one of the subcomponents with an increasing trend might contribute to the AMR more in the future and vice versa. For these reasons, a traditional nonstationary approach may not be appropriate for such systematic nonstationary characteristics.

We propose a design rainfall estimation approach with a nonstationary frequency analysis that separately treats the regions (e.g., East Asia) that are influenced by clearly different subcomponents for a better understanding of future climate change. Specifically, the current study aims to develop a novel approach in (1) partitioning extreme rainfall data (*PM*_*TOT*_) into TC (*PM*_*TC*_) and non-TC (*PM*_*NTC*_) rainfall with a recently developed technique by Son, *et al*.^31^ [details in supplementary material]; (2) obtaining two AMR series from the separated TC and non-TC rainfall data; (3) modeling the *PM*_*TC*_ and the *PM*_*NTC*_ series with nonstationary GEV (generalized extreme value) model [details in Methods] separately; and (4) integrating the fitted two nonstationary GEV models to obtain a design rainfall estimate to adapt the nonstationarities in the *PM*_*TC*_ and *PM*_*NTC*_ series. Details of the overall procedure are provided in the ‘Methods’ section. The proposed approach is then applied to every rainfall station in South Korea and 10 rainfall stations in the Tokyo region that are usually in the path of TC in Japan.

## Results

Among the East Asian countries, South Korea presents a serious seasonal rainfall deviation in that most of the annual average rainfall (approximately 66%, 854.4 out of 1,292 mm) occurs during the rainy season (June-September), which makes it vulnerable to flooding^1^. In addition, this region has experienced extreme rainfall events causing water-related disasters from the two subcomponents, TC and non-TC rainfall. Therefore, South Korea was selected as the study area. Additionally, we included the Tokyo region as another study area because numerous TCs in the WNP have passed through this region (Supplementary Table S3).

**Figure 1:**
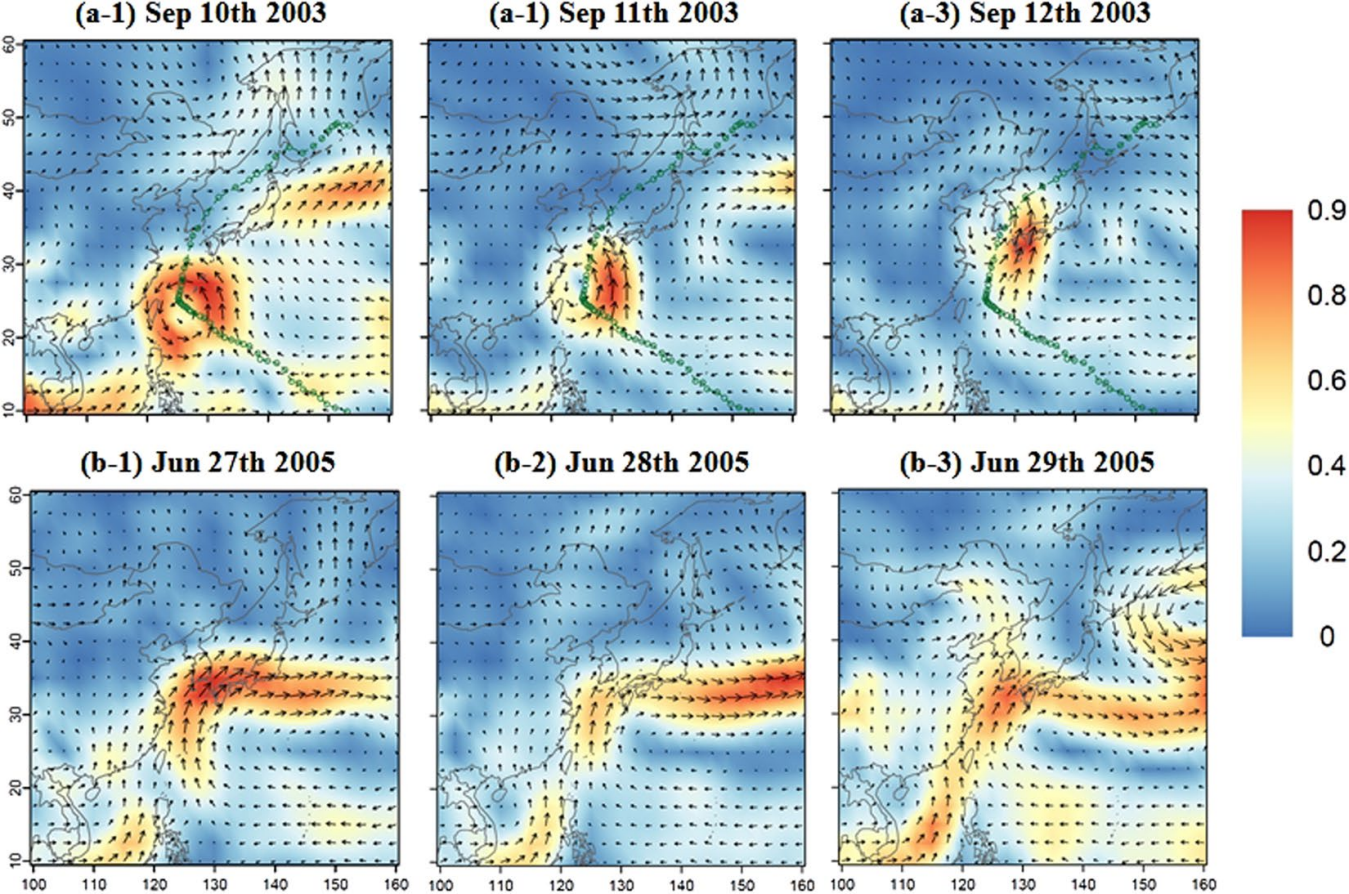
Two extreme rainfall events of the TC case (TC Maemi) in the top panels (**a-1**~**a-3**) and the non-TC case (monsoon rainfall) in the bottom panels (**b-1**~**b-3**). Note that blue to red shading represents the strength of VIMF (unit: g/kg · m/s); arrows indicate the mean wind vector over the vertical range between 1000 hPa and 750 hPa, and a green dotted line with circles in the top panels represents the TC path starting from the right bottom and heading northward. The maps were created using software “R studio Desktop, [1.0.44 version], (https://www.rstudio.com/products/rstudio/download3/)”.

Figure 1 shows the geographical distribution of a vertically integrated moisture flux (VIMF) [details in Methods]^32^ related to a TC rainfall event that occurred in September 2003 (Fig. 1(a)) and a non-TC rainfall event that occurred in June 2005 (Fig. 1(b)) over East Asia. Moreover, TCs commonly generate over the WNP in conditions of warm temperatures (above 26.5 °C), high relative humidity, and low vertical wind shear^33, 34^. After their formation, TCs accompanied by strong winds and high moisture content tend to move across the WNP Ocean and approach East Asia in an arched pattern along the edges of the North Pacific high air mass.

**Figure 2:**
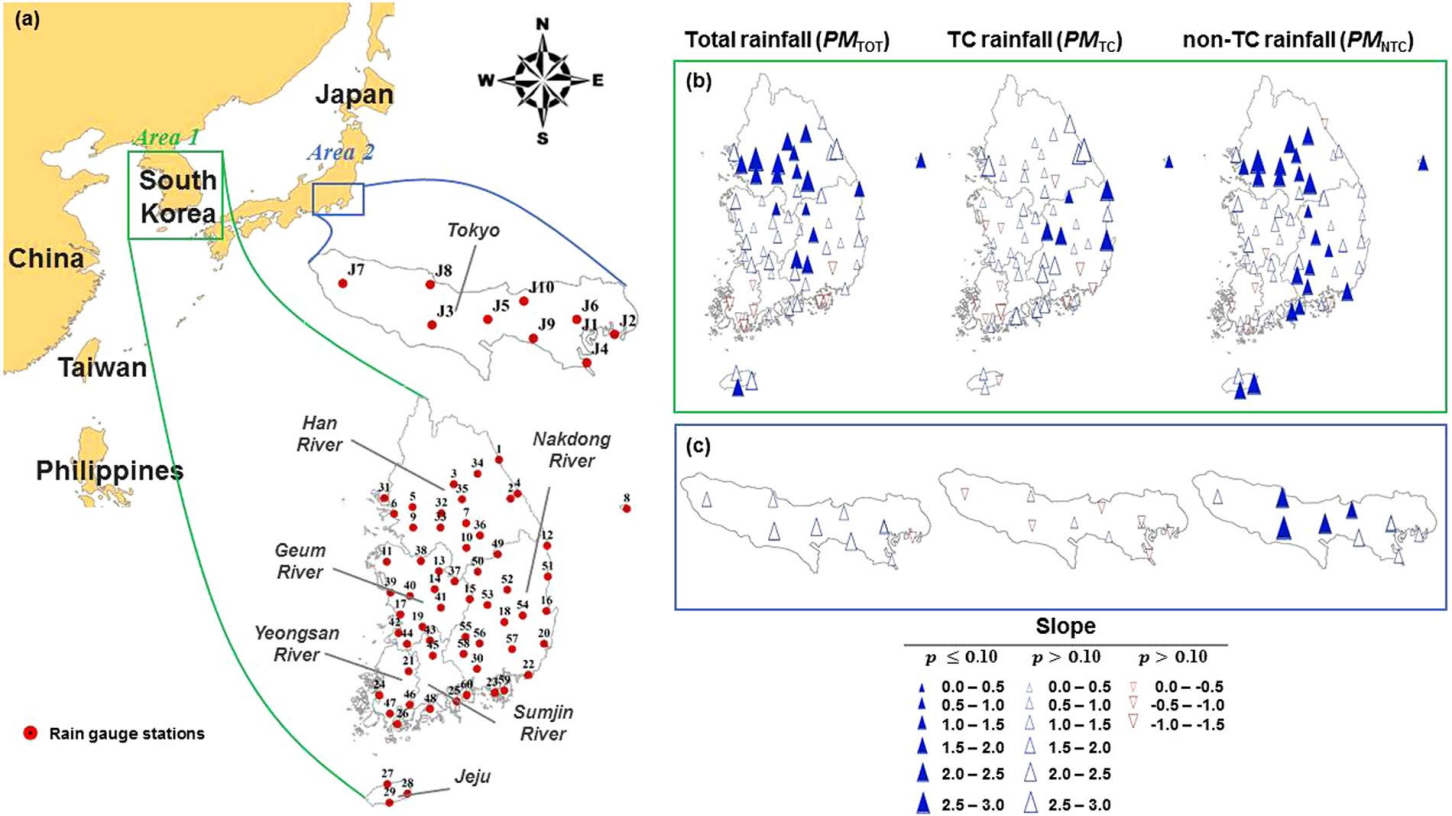
Geographical map of East Asia including locations of rain gauge stations in the two study areas (area 1: South Korea and area 2: Tokyo, Japan) (**a**) and trends in annual maximum daily rainfall as total rainfall, TC rainfall, and non-TC rainfall (**b**,**c**). Note that the Mann-Kendall statistical test is used to detect trends in a series; closed triangles represent significant trends at the 90% confidence levels. The maps were generated using software “ArcGIS 10.1 (ESRI, Redlands, CA, USA: http://www.esri.com/software/arcgis)”.

**Figure 3:**
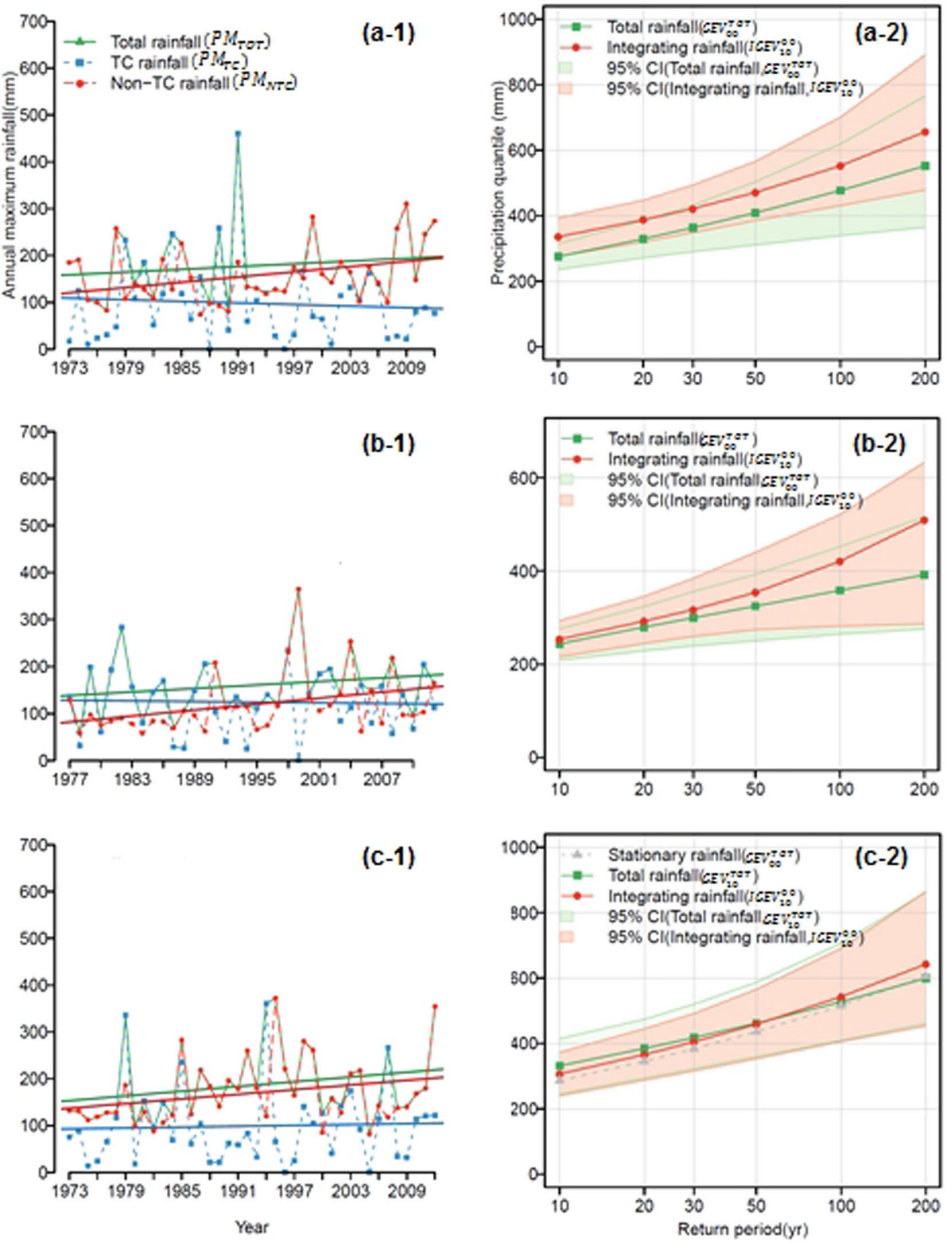
Time series of annual maximum daily rainfall of total rainfall (green dotted line with upward-pointing triangles; *PM*_*TOT*_), TC rainfall (blue dotted-dashed line with squares; *PM*_*TC*_), and non-TC rainfall (red dotted-dashed line with circles; *PM*_*NTC*_) for (a) Busan (station 22), (b) Hachioji (station J3), and (c) Seogwipo (station 29) in the left panels (**a-1**,**b-1** and **c-1**). The straight lines indicate the linear fit to annual maximum daily rainfall. The right panels (**a-2**,**b-2** and **c-2**) represent the quantiles with 95% confidence intervals estimated by the nonstationary *GEV* models between total rainfall (green dotted line with squares; (**a-2** and **b-2**) are $GE{V}_{00}^{TOT}$ models, and (**c-2**) is $GE{V}_{10}^{TOT}$) and integrated rainfall (red dotted line with circles; $IGE{V}_{10}^{00}$); the gray dotted-dashed lines with upward-pointing triangles in the c-2 indicate the quantiles estimated by the stationary *GEV* model ($GE{V}_{00}^{TOT}$). Note that (1) the confidence intervals were estimated with the bootstrapping method with 1000 resampling; (2) there are some cases that TC rainfall does not occur in a certain year (for example, seogwipo station at 1996, 2005 at (**c-1**)) and the occurrence probability is adopted in these cases as shown in Eqs (6) and (7) with *p*_*o*_ to take the non-occurrence of TC rainfall into account for quantile estimation.

For example, the TC Maemi that occurred on September 10–12, 2003, is presented in the top panels of Fig. 1. The TC originated over the WNP and headed northwestward toward Taiwan with intensifying magnitude on September 10, 2003, and hesitated near Taiwan (its center was located at 125°E, 25°N) as shown in Fig. 1(a-1). Afterward, the TC recurved northeastward toward the Korea Strait and the southern Sea of Japan on September 11 (see Fig. 1(a-2)). Eventually, the TC emerged on the Korean peninsula on September 12 (see Fig. 1(a-3)) and poured out tremendous amounts of rainfall, causing severe destruction of hydraulic structures and economic losses as well as 130 casualties.

A non-TC event (a typical case of monsoon rainfall, also called Changma in Korea) is a part of the quasi-stationary front of the East Asia summer monsoon season. For example, the non-TC event that occurred on June 27–29, 2005, is presented in the bottom panels of Fig. 1(b-1~b-3). The accumulation of moisture was maximized in the East China Sea and the WNP Ocean and traveled northeastward near the east side of Taiwan (Fig. 1(b-1)). The northeastward movement of the moisture mass was established by the development of the subtropical ridge located at the bottom right side of the moisture mass. Hence, the moisture was concentrated in the midlatitude regions (30–60°N) of the Northern Hemisphere including South Korea and Japan because of the subtropical environment (approximately 20–30°N, 130–160°E) and an Okhotsk high (approximately 40–50°N, 140–160°E) located in the southeast and northeast of the Korea peninsula, respectively (called Changma front). As shown in Fig. 1(b-1) and (b-3), a large mass of moisture constantly traveled toward the region containing Taiwan, South Korea, and Japan and persisted through July 13, 2005 (approximately three weeks). This monsoon rainfall (a typical severe event of a non-TC rainfall) was an intense and prolonged event triggering landslides and floods with 345 casualties as well as severe social and economic losses.

As Fig. 1 indicates, the characteristics of TC and non-TC rainfall events present a clear difference in terms of their intensity and duration. Therefore, it is apparent that the future trends of TC and non-TC rainfall events are possibly different, and an extreme analysis associated with them must be performed individually.

Therefore, a non-parametric Mann-Kendall trend test^35^ was used to assess the statistical significance of trends in different AMR series such as *PM*_*TOT*_, *PM*_*TC*_, and *PM*_*NTC*_. Figure 2(b) and (c) present the results of the Mann-Kendall test with a 10% significance level (*p* ≤ 0.1) for the *PM*_*TOT*_, *PM*_*TC*_, and *PM*_*NTC*_ series affecting South Korea and Tokyo. In South Korea, the *PM*_*TOT*_ series (the left side of Fig. 2(b)) tends to increase significantly over a part of South Korea including the Han River and Nakdong River basin (approximately 30% of the stations). For the *PM*_*TC*_ (the middle map of Fig. 2(b)), a significant and increasing trend can be observed in a part of the Nakdong River basin (approximately 10% of the stations). The *PM*_*TC*_ series exhibits stationary behavior for most areas of South Korea except for the Nakdong River basin.

In contrast, the *PM*_*NTC*_ series shows a clearly increasing trend in the Han River and Nakdong River basin (38% of the stations). In particular, the *PM*_*NTC*_ series shows a similar increasing trend as that observed in the *PM*_*TOT*_ series in the northwestern area of South Korea such as the Han River and Geum River basins. This result indicates that the *PM*_*TC*_ events have little effect on extreme rainfall; however, the *PM*_*NTC*_ series contributes to most of the AMR series (*PM*_*TOT*_) in the northwestern areas of South Korea (Han River: 87.3%; Geum River: 89.0%, the ratio of the *PM*_*NTC*_ in the *PM*_*TOT*_ series). In a part of the southern area of South Korea, the *PM*_*TOT*_ shows different tendencies in terms of sign (increase or decrease) and strength compared with those of the *PM*_*TC*_ and *PM*_*NTC*_ because the trends may be offset by the behaviors of the two subcomponents (TC and non-TC). Therefore, the trend assessment with *PM*_*TOT*_ may result in an unreliable future projection in the region where the AMR events (*PM*_*TOT*_) comprise the two subcomponents.

In the Tokyo region, the *PM*_*TOT*_ and *PM*_*TC*_ series show no significant trends even though an increasing trend is seen in the *PM*_*TOT*_ series. The *PM*_*TC*_ series does not show a significant trend over the region. However, the *PM*_*NTC*_ series increases significantly in the middle part of the Tokyo region (40% of the stations). Nonetheless, the *PM*_*NTC*_ series shows a significant trend, and this result suggests that the *PM*_*TOT*_ series for design rainfall can be treated as stationary. Therefore, the AMR series (*PM*_*TC*_ and *PM*_*NTC*_) must be treated differently when nonstationary processes are required and when extreme rainfall occurs due to two or more different weather systems.

The key features of the AMR series (*PM*_*TC*_ and *PM*_*NTC*_) over the study area that are identified in this study are as follows: First, the *PM*_*TC*_ series exhibits nearly stationary behavior, while the *PM*_*NTC*_ series appears to be nonstationary, showing noticeable trends. Second, although evidence for nonstationarity in the *PM*_*NTC*_ series is clear, there is, however, evidence of stationarity in the *PM*_*TOT*_ series. This may be interpreted as representing a stationary behavior buried in a nonstationary trend by compensating for the stationarity in the *PM*_*TC*_ series.

For evaluating the suitability of five different probability distributions as normal, Gamma, logistic, Gumbel, and GEV distributions, the Akaike Information Criterion (AIC)^36^, Bayesian Information Criterion (BIC)^37^, and Kolmogorov-Smirnov (K-S) test^38, 39^ can be used. Calculating the AIC and BIC for five distributions, we found that the Gamma, gumbel, and GEV distributions are selected for a number of rainfall stations (Supplementary Fig. S2). In the two study areas, the frequency analysis of extreme rainfall events often apply the Gumbel and GEV distributions in literature. In addition, Wi, *et al*.^29^ suggested that the GEV distribution appropriately represents the AMP series for South Korea. In particular, the GEV distribution is more flexible relative to the Gumbel distribution because of using a larger number of parameters^40^. Overall, K-S test justified the use of the GEV distribution as an appropriate alternative for all stations in the two study areas. Therefore, the GEV distribution is used to fit the AMR series in the current study.

Figure 3 presents the time series of the AMR series (*PM*_*TOT*_, *PM*_*TC*_, and *PM*_*NTC*_) with linear slopes (left panels) and the rainfall quantile estimates with 95% confidence intervals (right panels) for the selected and stationary *GEV* models using the total rainfall (*GEV* ^*TOT*^ model) and integrated rainfall (*IGEV* model) for the selected sites of Busan (a), Hachioji (b), and Seogwipo (c). Details of the stationary or nonstationary GEV models are described in the ‘Methods’ section. To estimate confidence intervals for the parameters (Supplementary Fig. S3) and quantiles (Fig. 3) of selected stationary and nonstationary GEV models using the total rainfall and integrated rainfall, the bootstrap method was used. In the current study, 1000 bootstrap samples were generated from fitted selected GEV models. For example, in Fig. 3(a-2), the stationary model is used to fit the *PM*_*TOT*_ series of Busan (station no. 22, see Fig. 2(a)) since the Mann-Kendall test shown in Fig. 2(b) has no significant trends. Meanwhile, the separate consideration of the *PM*_*TC*_ and the *PM*_*NTC*_ series by integrating the two series through equation (7) leads to the nonstationary model of $IGE{V}_{10}^{00}$ for reflecting the identified trends in the *PM*_*TC*_ (i.e., stationarity) and the *PM*_*NTC*_ (i.e., nonstationarity), as shown in Fig. 3(a-1).

The stationary model ($GE{V}_{00}^{TOT}$) for the *PM*_*TOT*_ series of the Busan and Hachioji stations is illustrated in Fig. 3(a-1) and (b-1). Note that one of the *PM*_*TC*_ and *PM*_*NTC*_ values is selected over time for each value of the *PM*_*TOT*_ series such that the *PM*_*TOT*_ value overlaps with one of the *PM*_*TC*_ or the *PM*_*NTC*_ values, as shown in Fig. 3(a1). The *PM*_*TOT*_ series of the Busan (a-1) and Hachioji (b-1) stations show that the *PM*_*TC*_ are generally selected for the *PM*_*TOT*_ series during the early time periods (i.e., 1973–1995), while for the latter periods (i.e., 1996–2012), the *PM*_*NTC*_ is more prevalent. As aforementioned, this is mainly due to different levels of nonstationarity over time in the two time series. For these reasons, the estimated design rainfalls may not be reliable as shown in the right panels of Fig. 3(a-2) and (b-2) without treating the AMR series separately. The *IGEV* model shows higher values than the stationary model for all the return periods, and more specifically, higher return periods shows higher differences. In this setting, the stationary model for critical hydraulic structures (e.g., dams and large river levees) associated with high return periods can lead to an underestimation of hydrologic risk.

As represented in Fig. 3(c), there are nonstationary behaviors of the *PM*_*TOT*_ and *PM*_*NTC*_ series, while the *PM*_*TC*_ series presents stationary behavior for Seogwipo (i.e., station no. 29). Although the y-intercept of the linear fit from the *PM*_*TOT*_ series is higher than that of the *PM*_*NTC*_, both have similar slopes to the *PM*_*TOT*_: 1.688/year and *PM*_*NTC*_: 1.657/year, which are statistically significant. Figure 3(c-2) shows the differences between quantiles of the $GE{V}_{10}^{TOT}$ model and $IGE{V}_{10}^{00}$ model. For higher return periods, the quantiles of the $IGE{V}_{10}^{00}$ model tend to be larger than those of the $GE{V}_{10}^{TOT}$ model because the $GE{V}_{10}^{TOT}$ model could not consider the enhanced variability in the *PM*_*TC*_ series (arithmetic standard deviations of the *PM*_*TC*_ and *PM*_*NTC*_ are 65.84 mm and 52.33 mm).

In other words, higher scale parameter (Supplementary Fig. S3(b-2) and (c-2)) obtained from *PM*_*TC*_ series may lead to a relatively higher quantile for higher return periods and vice versa while smaller location parameter of non-TC rainfall than total rainfall as shown in Fig. 3(c-2) may lead to relatively smaller quantiles during lower return periods. In terms of safety, it means that the proposed approach could be better than the existing nonstationary approach at estimating design rainfalls for dam structures with the high return periods.

**Figure 4:**
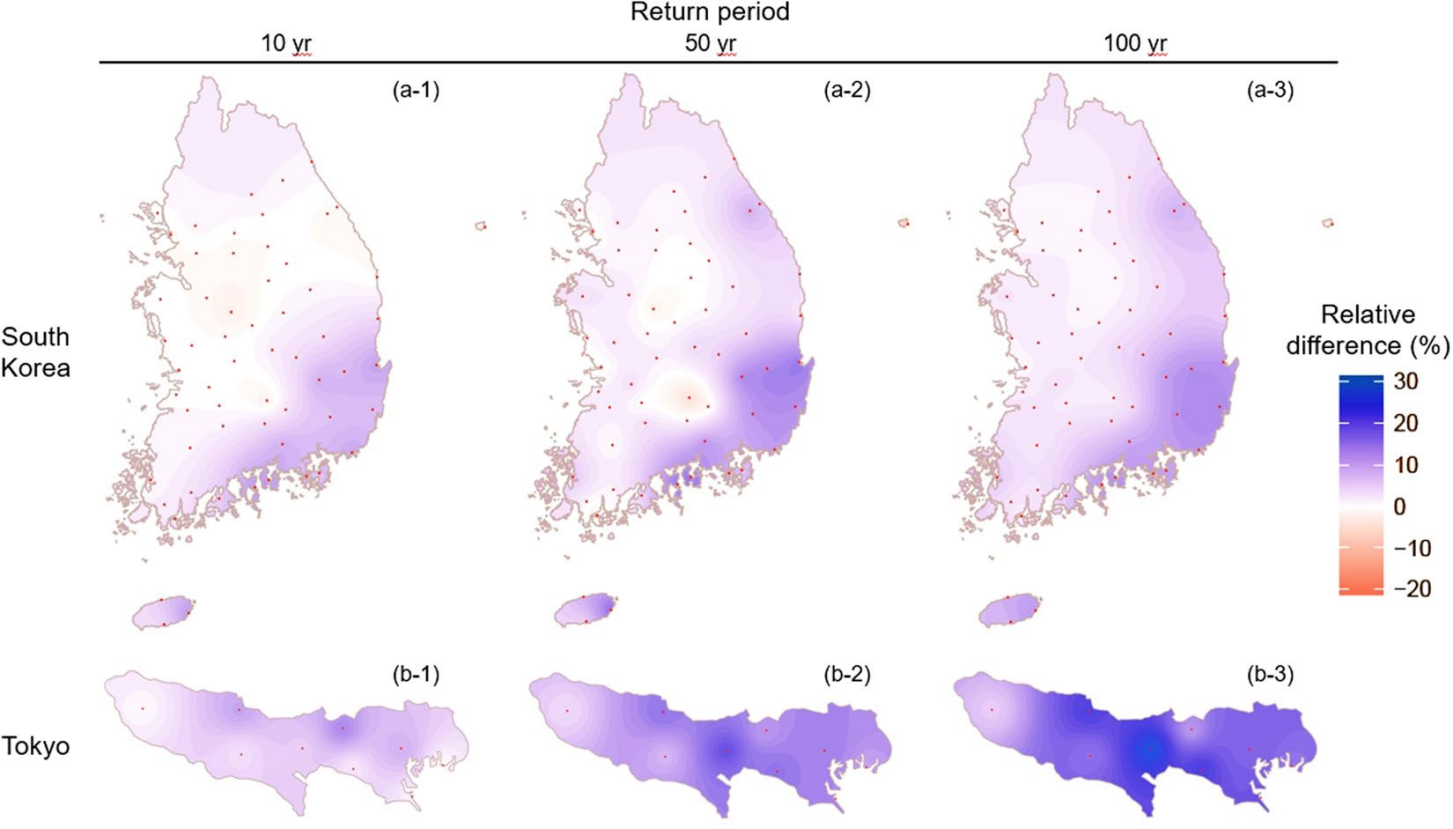
Relative percent differences (RPD = (*IGEV* − *GEV* ^*TOT*^)/*GEV* ^*TOT*^, %) of the quantiles estimated with the nonstationary *GEV* model between total rainfall (*GEV* ^*TOT*^) and integrated rainfall (*IGEV*) for the 10-, 50-, and 100-yr return period over (a) South Korea and (b) Tokyo. Note that the Kriging method is used for interpolation; blue (red) shading indicates that the quantiles estimated with the *IGEV* model are larger (smaller) than the quantiles estimated with the *GEV* ^*TOT*^ model, and red points indicate rain gauge stations. The map were generated using software “R studio Desktop, [1.0.44 version], (https://www.rstudio.com/products/rstudio/download3/)”.

To quantitatively identify the spatial features of design rainfalls with different return levels, the relative percent differences (RPD) were calculated as the differences of *IGEV* − *GEV* ^*TOT*^ divided by *GEV* ^*TOT*^ model and expressed as a percentage. Figure 4 presents the RPD of the quantiles estimated by the *GEV* models for the three different return levels (i.e., 10, 50, and 100 years). In South Korea, the quantiles corresponding to the 10- and 50-yr return periods estimated by the *IGEV* model are higher than those estimated by the *GEV* ^*TOT*^ model especially in the southeast region of South Korea (Fig. 4(a-1) and (a-2)). In Tokyo, the quantiles estimated by the *IGEV* model shows higher values than those estimated by the *GEV* ^*TOT*^ model.

In general, the northern area of South Korea tends to be affected by extreme rainfall from only non-TC rainfall because numerous TCs move toward the southeast region of South Korea and the southern Sea of Japan. In the northwestern area including the Han River basin and Geum River basin, the quantiles estimated by the *IGEV* model are quantitatively similar to those estimated by the *GEV* ^*TOT*^ model. In contrast, in the southern area of South Korea and Tokyo, which are simultaneously influenced by the two subcomponents, the quantiles estimated by the *IGEV* and *GEV* ^*TOT*^ models show clear differences. These results indicate that the proposed approach can estimate relatively well by capturing nonstationary behaviors of non-TC rainfall better.

## Discussion

In this study, we proposed a novel method of integrating the fitted AMR series of TC and non-TC rainfall by nonstationary models. In South Korea, the *PM*_*TOT*_ and *PM*_*NTC*_ series exhibited statistically significant increases over the Han River basin and the western Nakdong River basin. However, no significant trends were found in *PM*_*TC*_ series, except for a statistically significant increasing trend at six stations in the Nakdong River basin. In Tokyo, the *PM*_*TOT*_ and *PM*_*TC*_ series showed no significant trends either. In contrast, the *PM*_*NTC*_ observed a statistically significant increase over the middle areas of Tokyo. It indicated that the future long-term evolutions of the two subcomponents might be different from each other. Therefore, TC and non-TC rainfall events must be treated differently when nonstationary frequency analyses are required and when extreme rainfall occurs due to two or more different weather systems.

To treat the nonstationarity of the AMR series differently, we carried out a nonstationary frequency analysis of the different AMR series from TC and non-TC events individually and integrated the fitted *PM*_*TC*_ and *PM*_*NTC*_ series with the developed *IGEV* model in the current study. We found different features over South Korea and Tokyo according to extreme events such as the *PM*_*TC*_ in stationary behavior and the *PM*_*NTC*_ in nonstationary behavior. However, it was not possible to consider the existing frequency analysis to be different in the long-term trends of the AMR series from the two subcomponents. Therefore, the existing stationary approach might have led to imprecise inferences because it ignored the nonstationarity of the non-TC rainfall. It was concluded that the proposed approach can be a useful alternative in designing hydraulic structures and can ensure safety against flood damage in a nonstationary regime of extreme rainfall events.

Only the time variable was considered as a covariate in mapping extreme rainfall to highlight the need to handle the nonstationarity of the AMR series from the two subcomponents separately. The results of the proposed approach were expected to provide a more reliable estimate for designing flood-related hydraulic structures considering climate change and the management of water resources. One can extend the current study to include the variability change, but sufficient number of AMR data must be obtained in advance. Sufficient evidences such that different weather systems lead to different behaviors for overall trends of AMR series must be provided to apply the proposed method.

## Methods

### Integrating procedure for TC and non-TC rainfall

A radius-based TC rainfall extraction method (RTREM) [details in supplementary material] was developed to differentiate series of rainfall into TC and non-TC rainfall. Among TC rainfall values, *X*_*t*_ was the annual maximum value at year *t*, and for non-TC rainfall, *Y*_*t*_ was used. The time index *t* was dropped for simplicity, and *X* and *Y* were used for the AMR series of TC and non-TC rainfall, respectively. Eventually, an AMR value for a certain year (*Z*_int_) was obtained by selecting the maximum value of *X* and *Y*.

1 $${Z}_{{\rm{int}}}=\,{\rm{\max }}(X,Y)$$

Note that *Z*_int_ indicated the integration of the two AMR series (*X* and *Y*). From a general statistical derivation, it was easily shown that

2 $${F}_{{Z}_{{\rm{int}}}}(z)=P({Z}_{{\rm{int}}} < z)=P({\rm{\max }}(X,Y) < z)=P(X < z,Y < z)$$

Since it was generally agreed upon that the AMR of the TC (*X*) and non-TC (*Y*) rainfall events were independent, equation (3) became

3 $${F}_{{Z}_{{\rm{int}}}}(z)=P(X < z,Y < z)=P(X < z)\cdot P(Y < z)={F}_{X}(z)\cdot {F}_{Y}(z)$$

The CDF of non-TC (*F*_*Y*_(*z*)) rainfall was denoted as

4 $${F}_{Y}(z)=\exp \{-{(1-{\kappa }_{Y}(t)\frac{z-{\mu }_{Y}(t)}{{\alpha }_{Y}(t)})}^{1/{\kappa }_{Y}(t)}\}$$

Furthermore, TC may not have occurred every year in a target area. Therefore, its occurrence was adapted as

5 $${p}_{o}={n}_{TC}/n$$

where *p*_*o*_ was the probability that TC occurred in a certain year; *n*_*TC*_ was the number of years that TC occurred, and *n* was the number of record years. Therefore, the CDF of TC was denoted as

6 $${F}_{X}(z)=(1-{p}_{o})+{p}_{o}\exp \{-{(1-{\kappa }_{X}(t)\frac{z-{\mu }_{X}(t)}{{\alpha }_{X}(t)})}^{1/{\kappa }_{X}(t)}\}$$

Finally, the CDF of *Z*_*int*_ in equation (1) was described as

7 $$\begin{array}{rcl}{F}_{{Z}_{{\rm{int}}}}(z) & = & [(1-{p}_{o})+{p}_{o}\exp \{-{(1-{\kappa }_{X}(t)\frac{z-{\mu }_{X}(t)}{{\alpha }_{X}(t)})}^{1/{\kappa }_{X}(t)}\}]\\  &  & \cdot \exp \{-{(1-{\kappa }_{Y}(t)\frac{z-{\mu }_{Y}(t)}{{\alpha }_{Y}(t)})}^{1/{\kappa }_{Y}(t)}\}\end{array}$$

### Vertically Integrated Moisture Flux (VIMF)

The VIMF presented in Fig. 1 was calculated by integrating wind vector and specific humidity from 1000 to 850 hPa (approximately 1.5 km) as

8 $$VIMF=\Vert {W}_{u}\times SH+{W}_{v}\times SH\Vert $$

where *W*_*u*_ and *W*_*v*_ denoted the average wind vector in the x- and y-directions for the range 1000 and 850 hPa, respectively, and *SH* was the integrated specific humidity for the same range. The VIMF provided succinct information on the atmospheric moisture cycle.

### Procedure for the quantile estimation

A detailed description of the modeling procedure is as follows (Supplementary Fig. S1):

1. Extract TC rainfall events (*P*_*TC*_) among the total rainfall events (*P*_*TOT*_) every year with RTREM. Let the remainder be non-TC rainfall (*P*_*NTC*_).
2. Obtain the AMR series including the *PM*_*TC*_, *PM*_*NTC*_, and *PM*_*TOT*_ series from rainfall datasets (*P*_*TOT*_, *P*_*TC*_, and *P*_*NTC*_).
3. Fit a nonstationary *GEV* model [details in supplementary material] to the obtained *PM*_*TOT*_, *PM*_*TC*_, and *PM*_*NTC*_ individually.
4. Integrate the fitted series of *PM*_*TC*_ and *PM*_*NTC*_ with equation (7).
5. Estimate the quantiles of the interested return periods (*T*_*R*_) such as 10, 20, 30, 50, 100, 200 years. Note that a quantile can be estimated with a known *T*_*R*_ and its corresponding CDF as *F* = 1 − (1/*T*_*R*_). The quantile value (*z*) in equation (7) can be numerically calculated with the same equation since all the other parameters are already estimated.

Numerous nonstationary *GEV* models were applied in the current study according to the location and scale parameters. We denoted the model $GE{V}_{ij}^{m}$ with the order *i* for the location parameter and the order *j* for the scale parameter, while the superscript *m* indicated the type of rainfall datasets such as total (*TOT*) rainfall, TC (*TC*) rainfall, or non-TC (*NTC*) rainfall. We restricted the model to cases of nonstationarity in the location and scale parameters and expressed it as follows:

1. $GE{V}_{00}^{TOT}(\mu ,\alpha ,\kappa )$, $GE{V}_{00}^{TC}({\mu }_{X},{\alpha }_{X},{\kappa }_{X})$, or $GE{V}_{00}^{NTC}({\mu }_{Y},{\alpha }_{Y},{\kappa }_{Y})$ represented the stationary model as all parameters were assumed to be constant: *μ*(*t*) = *μ*, *α*(*t*) = *α*, *κ*(*t*) = *κ*.
2. $GE{V}_{10}^{TOT}(\mu (t)={\beta }_{0}+{\beta }_{1}t,\alpha ,\kappa )$, $GE{V}_{10}^{TC}({\mu }_{X}(t),{\alpha }_{X},{\kappa }_{X})$, or $GE{V}_{10}^{NTC}({\mu }_{Y}(t),{\alpha }_{Y},{\kappa }_{Y})$ represented the nonstationary model with the time-varying location parameter that was linearly dependent on time.
3. $GE{V}_{11}^{TOT}(\mu (t)={\beta }_{0}+{\beta }_{1}t,\alpha (t)=\exp ({\delta }_{0}+{\delta }_{1}t),\kappa )$, $GE{V}_{11}^{TC}({\mu }_{X}(t),{\alpha }_{X}(t),{\kappa }_{X})$, or $GE{V}_{11}^{NTC}({\mu }_{Y}(t),$ ${\alpha }_{Y}(t),{\kappa }_{Y})$ indicated the nonstationary model with trends in both location and scale parameters. Note that the generalized maximum likelihood (GML) approach is used to estimate the parameters in GEV distribution.
4. $IGE{V}_{lm}^{ij}$ was the integrated model of $GE{V}_{ij}^{TC}$ and $GE{V}_{lm}^{NTC}$ as in Equation (7) where *i* and *j* indicated the orders of the nonstationary GEV model for TC rainfall, while *l* and *m* represented the orders of the nonstationary GEV model for non-TC rainfall. For example, $IGE{V}_{10}^{00}$ represented the integrated model of the stationary GEV model for TC rainfall ($GE{V}_{00}^{TC}$) and the linear model of the location parameter for non-TC rainfall ($GE{V}_{10}^{NTC}$).

### Data sets

- *Rainfall data*. Hourly rainfall data was obtained from 60 rain gauge stations in South Korea and 10 rain gauge stations in the Tokyo region (Fig. 2(a)). These data from South Korea during the period of 1973–2012 were provided by the Korean Meteorological Administration (http://www.kma.go.kr). For the data from the Tokyo region, hourly rainfall data for the period of 1977–2012 were obtained from the Japan Meteorological Agency (http://www.jma.go.jp).
- *Tropical cyclone (TC) data*. Information about the TCs that occurred in the WNP over a 40-year period, spanning from 1973 to 2012, was obtained from the Tokyo-Typhoon Center. The acquired TC information included wind speed, central pressure and location of the TC center for every six hours. Accordingly, the TC rainfall amount was extracted with the RTREM. The probability of a TC occurrence each year in equation (5) was estimated from this information.

## Electronic supplementary material

Supplementary Info
