# Supplementary material for: Integrating nonstationary behaviors of typhoon and non-typhoon extreme rainfall events in East Asia
Source: Sci Rep. 2017 Jul 11;7:5097. doi: 10.1038/s41598-017-04629-1 (PMC5506020; doi:10.1038/s41598-017-04629-1)
Supplement: Supplementary file 1 — Supplementary Info [file 41598_2017_4629_MOESM1_ESM.pdf]

Supporting Online Material for:

Integrating nonstationary behaviors of typhoon and non-typhoon extreme rainfall events in East Asia

Chanyoung Son[1], Taesam Lee[1*], and Hyunhan Kwon[2]

[1] Department of Civil Engineering, ERI, Gyeongsang National University, 501 Jinju-daero, Jinju, Gyeongsangnam-do, South Korea

[2] Department of Civil Engineering, Chonbuk National University, Deokjin-dong 1ga, Deokjin-gu, Jeonju-si, Jeollabuk-do, South Korea

**Correspondence to: Taesam Lee (tae3lee@gnu.ac.kr)*

Supplementary Text

Nonstationary GEV model

In extreme value theorem, the maxima of sequences of identically and independently distributed random variables can only converge to the GEV distribution [S1]. Due to its role, the GEV distribution has been widely used for frequency analysis of hydro-meteorological variables [S2-S4]. However, hydrologic variables such as rainfall and flood exhibit nonstationary behavior and show some evidence of quasiperiodic trends as well as long range time dependence in recent years due to either climate variability or change. In this context, the parameters of the GEV distribution may be treated as nonstationary [S5-S9]. The general form of the GEV distribution and its nonstationary form can be described as follows:

The cumulative distribution function (CDF) of the GEV distribution, introduced by Jenkinson [S10], is given by:

$$F(z;\mu,\alpha,\kappa)=\exp\left\{-\left(1-\kappa\frac{z-\mu}{\alpha}\right)^{1/\kappa}\right\},\kappa\neq 0 \text{ and } 1-\kappa\frac{(z-\mu)}{\alpha}>0 \qquad \text{(S1)}$$

where μ, α, and κ are the location, scale, and shape parameters and $z=\{z(t),t=1,...,n\}$ is a series of independent observations. For estimation of the parameters in traditional GEV distribution, the maximum likelihood (ML) approach is generally used, but its estimates are often unstable because of the estimation of unrealistic shape parameter values (κ) for small sample sizes [S11,S12]. Martins and Stedinger [S12] proposed an improved ML approach so that the shape parameter is forced to take more realistic value, named as the generalized maximum likelihood (GML) approach, which applies a more restrictive penalty using a beta distribution as prior for the shape parameter, leading to

$$L^g(\mu,\alpha,\kappa|z)=L(\mu,\alpha,\kappa|z)\pi^g(\kappa) \qquad \text{(S2)}$$

$$\pi^g(\kappa) = Beta(\kappa + 0.5; c_1, c_2) \tag{S3}$$

where $L^g(\mu, \alpha, \kappa | z)$ and $L(\mu, \alpha, \kappa | z)$ are the likelihood functions for the GML and standard approach, while $\pi^g(\kappa)$ is a penalty (or prior distribution) for the shape parameter.

A number of literature reported that the GML approach presents better estimation for the nonstationary GEV model[S5,S7,S12,S13]. Therefore, the GML approach is used to estimate GEV parameters in the current study.

In the nonstationary case, the parameters in GEV distribution are expressed as a function of covariates such as time itself or any other time-varying variables. The CDF of nonstationary GEV model is determined as:

$$F(z; \mu(t), \alpha(t), \kappa(t)) = \exp\left\{ -\left(1 - \kappa(t) \frac{z - \mu(t)}{\alpha(t)}\right)^{1/\kappa(t)} \right\} \tag{S4}$$

The logarithm of the scale parameter is, in general, used to ensure its value positive. To account for a linear or nonlinear trend in GEV distribution, the general form of three parameters can be written as follows:

$$\mu(t) = \beta_0 + \beta_1 t + \beta_2 t^2 + \cdots, \tag{S5}$$

$$\ln[\alpha(t)] = \delta_0 + \delta_1 t + \delta_2 t^2 + \cdots, \tag{S6}$$

$$\kappa(t) = \gamma_0 + \gamma_1 t + \gamma_2 t^2 + \cdots \tag{S7}$$

where t is time or any other time-varying variables, $\mu(t)$, $\alpha(t)$, and $\kappa(t)$ are the location, scale, and shape parameter of nonstationary case at time t, and β, δ, and γ are the regression coefficients for the time-varying variables.

In this current study, the nonstationarity of the shape parameter in equation (S4) is not considered ($\kappa(t) = \kappa$) because the use of short record length (about 30-50years) can result in less reliable estimates in shape parameter [S12, S14-S16].

Radius-based TC rainfall extraction method (RTREM)

TC rainfall should be properly defined in order to better identify and deal with nonstationarities in TC and non-TC rainfall, separately. A domain based approach was employed to extract TC rainfall events from total rainfall events, which determines a TC rainfall event in case that a TC influences the subjectively pre-determined domain surrounding an interested area [S17,S18]. However, this approach has a drawback such that the TC rainfall cannot be correctly detected, because it accounts for the case in which the center of TC is in the domain.

To remedy this drawback, Son et al. [S19] proposed an approach determining the TC rainfall from total rainfall data, called a radius-based TC rainfall extraction method (RTREM) and defined TC rainfall as a measured rainfall from a station within 700km radius from TC center. In addition, Lee et al.[S20] defined TC-induced rainfall as station rainfall within a 5˚ radius from TC center. Because radii defined from previous studies for extracting TC rainfall are different, the current study compared and reviewed the quantitative features of the TC rainfall for different TC radii (500-700km) focusing on the TCs accompanied by heavy rainfall such as Rusa, Agnes, Gladys, and Nari.

Supplementary Table S1 and S2 present the results of extracted TC rainfall for 1-hr and 24-hr durations according to different radii. Note that the reference value is TC rainfall extracted with checking station rainfall one-by-one. For 1-hr duration (Supplementary Table S1), TC rainfall extracted by using 500 or 600 km radius is less

than or equal to reference value while those by using 700km radius is equal to the reference value. For 24-hr duration (Supplementary Table S2), there are significant difference between TC rainfall extracted by 500 or 600 km radius and the reference value for Rusa, Agnes, and Nari while the TC rainfall extracted by 700 km is equal to reference value except for Rusa. These results indicate that setting of TC radius is important for extracting more accurate intensities and amounts of TC rainfall and 700km radius defined from Son et al.[S19] is appropriate. Therefore, the approach proposed by Son et al. [S19] was mainly utilized in the current study to separate TC and non-TC rainfall data. A detailed description of the RTREM procedure is as the following :

(1) Interpolate 6 hourly TC track data (TC_k^{6hr}, $k = 1,2,...N_{TC}$ where N_{TC} is the number of TCs in the western North Pacific) to hourly data (TC_k^{1hr}) because hourly TC track data is required to match up to the hourly rainfall data.

(2) Estimate the distance between the center of TC and the rain gauge locations inside a target area ($D_i, i = 1,2 \cdots n_g$ where n_g is the number of rain gauges).

(3) Select TC events affecting the target area with the condition that any distances (D_i) are smaller than an assumed TC radius between 100 and 1000km with 100km increment (R_j, j=100, 200, $\cdots$, 1000km) and obtain the rainfall data whose rain gauge is inside the assumed TC radius.

(4) Calculate the mean of the obtained rainfall data for each assumed R_j.

(5) Determine an optimal TC radius that provides the maximum of the mean of TC rainfall of all stations among the R_j.

(6) Extract TC rainfall from total rainfall series when the rain gauge is inside the optimal TC radius. Note that the remaining rainfall dataset excluding TC rainfall from total rainfall series is referred to as non-TC rainfall.

Supplementary Reference

S1. Coles, S., J. Bawa, L. Trenner, and P. Dorazio (2001), *An introduction to statistical modeling of extreme values*, Springer.

S2. Cannon, A. J. (2015), An intercomparison of regional and at-site rainfall extreme value analyses in southern British Columbia, Canada, *Canadian Journal of Civil Engineering*, *42*(2), 107-119.

S3. Cheng, L., A. AghaKouchak, E. Gilleland, and R. W. Katz (2014), Non-stationary extreme value analysis in a changing climate, *Climatic change, 127*(2), 353-369.

S4. Salas, J. D., and J. Obeysekera (2013), Revisiting the concepts of return period and risk for nonstationary hydrologic extreme events, *Journal of Hydrologic Engineering*, *19*(3), 554-568.

S5. Cannon, A. J. (2010), A flexible nonlinear modelling framework for nonstationary generalized extreme value analysis in hydroclimatology, *Hydrological Processes*, *24*(6), 673-685.

S6. Cunderlik, J. M., and D. H. Burn (2003), Non-stationary pooled flood frequency analysis, *Journal of Hydrology*, *276*(1), 210-223.

S7. El Adlouni, S., T. B. M. J. Ouarda, X. Zhang, R. Roy, and B. Bobée (2007), Generalized maximum likelihood estimators for the nonstationary generalized extreme value model, *Water Resources Research*, *43*(3).

S8. Leclerc, M., and T. B. Ouarda (2007), Non-stationary regional flood frequency analysis at ungauged sites, *Journal of Hydrology*, *343*(3), 254-265.

S9. Panagoulia, D., P. Economou, and C. Caroni (2014), Stationary and nonstationary generalized extreme value modelling of extreme precipitation over a mountainous area under climate change, *Environmetrics*, *25*(1), 29-43.

S10. Jenkinson, A. F. (1955), The frequency distribution of the annual maximum (or minimum) values of meteorological elements, *Quarterly Journal of the Royal Meteorological Society*, *81*(348), 158-171.

S11. Coles, S., and M. J. Dixon (1999), Likelihood-based inference for extreme

value models, *Extremes*, *2*(1), 5-23.
S12. Martins, E. S., and J. R. Stedinger (2000), Generalized maximum-likelihood generalized extreme-value quantile estimators for hydrologic data, *Water Resources Research*, *36*(3), 737-744.
S13. Kyselý, J. and J. Picek (2007), Regional growth curves and improved design value estimates of extreme precipitation events in the Czech Republic, *Climate Research*, 33, 243-255.
S14. Koutsoyiannis, D. (2004), Statistics of extremes and estimation of extreme rainfall: II. Empirical investigation of long rainfall records/Statistiques de valeurs extrêmes et estimation de précipitations extrêmes: II. Recherche empirique sur de longues séries de précipitations, *Hydrological Sciences Journal*, *49*(4), 591-610.
S15. Ouarda, T. B. M. J., and S. El-Adlouni (2011), Bayesian nonstationary frequency analysis of hydrological variables1, *Journal of The American Water Resources Association*, *47*(3), 496-505.
S16. Vasiliades, L., P. Galiatsatou, and A. Loukas (2015), Nonstationary frequency analysis of annual maximum rainfall using climate covariates, *Water Resources Management*, *29*(2), 339-358.
S17. Kim, J., and S. Jain (2011), Precipitation trends over the Korean peninsula: typhoon-induced changes and a typology for characterizing climate-related risk, *Environmental Research Letters*, *6*(3), 034033.
S18. Son, C.-Y., J.-S. Kim, Y.-I. Moon, and J.-H. Lee (2014a), Characteristics of tropical cyclone-induced precipitation over the Korean River basins according to three evolution patterns of the Central-Pacific El Nino, *Stochastic environmental research and risk assessment*, *28*(5), 1147-1156.
S19. Son, C.-Y., H.-H. Kwon, J.-S. Kim, and Y.-I. Moon (2014b), Comparative Assessment of a Method for Extraction of TC-induced Rainfall Affecting the Korean Peninsula, *Journal of the Korean Water Resources Association Vol*, *47*(12), 1187-1198.
S20. Lee, M. H., C. H. Ho, J. H. Kim, and H. J. Song (2012), Low-frequency variability of tropical cyclone-induced heavy rainfall over East Asia associated with tropical and North Pacific sea surface temperatures, *Journal of Geophysical Research*, 117, D12101, doi:10.1029/2012JD017565.

Supplementary Table

Supplementary Table S1. Maximum TC rainfall amounts (mm) for 1-hr duration with the different radii (500-700km) focusing on the TCs that are accompanied by extremely heavy rainfall such as Rusa, Agnes, Gladys, and Nari. Note that reference value is TC rainfall extracted with checking the station rainfall one-by-one..

TC	Station		TC rainfall(mm)						
			Reference	Radius(km)			Difference		
	Name	No	value (①)	500(②)	600(③)	700(④)	②-①	③-①	④-①
RUSA(0215)	Gangneung	4	98.0	98.0	98.0	98.0	0.0	0.0	0.0
	Daegwallyeong	2	60.5	50.0	50.0	60.5	-10.5	-10.5	0.0
	Goheung	48	81.0	81.0	81.0	81.0	0.0	0.0	0.0
	Seongsan	28	22.5	22.5	22.5	22.5	0.0	0.0	0.0
AGNES(8118)	Ulleungdo	8	37.5	37.5	37.5	37.5	0.0	0.0	0.0
	Uljin	12	22.8	20.2	22.8	22.8	-2.6	0.0	0.0
	Mokpo	24	38.4	38.4	38.4	38.4	0.0	0.0	0.0
	Jangheung	46	52.5	52.5	52.5	52.5	0.0	0.0	0.0
	Jeju	27	71.0	71.0	71.0	71.0	0.0	0.0	0.0
GLADYS(9112)	Daegwallyeong	2	24.3	24.3	24.3	24.3	0.0	0.0	0.0
	Uljin	12	39.5	39.5	39.5	39.5	0.0	0.0	0.0
	Pohang	16	24.6	24.6	24.6	24.6	0.0	0.0	0.0
	Ulsan	20	34.1	34.1	34.1	34.1	0.0	0.0	0.0
	Busan	22	37.7	37.7	37.7	37.7	0.0	0.0	0.0
NARI(0711)	Jeju	27	70.5	70.5	70.5	70.5	0.0	0.0	0.0
	Seongsan	28	34.0	26.5	34.0	34.0	-7.5	0.0	0.0
	Goheung	48	90.5	90.5	90.5	90.5	0.0	0.0	0.0
	Namhae	60	48.0	48.0	48.0	48.0	0.0	0.0	0.0

Supplementary Table S2. Same as in Supplementary Table S1 but for 24-hr duration.

TC	Station		TC rainfall(mm)						
			Reference	Radius(km)			Difference		
	Name	No	value(①)	500(②)	600(③)	700(④)	②-①	③-①	④-①
RUSA(0215)	Gangneung	4	880.0	545.0	671.0	856.0	-335.0	-209.0	-24.0
	Daegwallyeong	2	718.0	434.5	507.0	697.0	-283.5	-211.0	-21.0
	Goheung	48	409.5	409.5	409.5	409.5	0.0	0.0	0.0
	Seongsan	28	136.5	136.5	136.5	136.5	0.0	0.0	0.0
AGNES(8118)	Ulleungdo	8	258.7	184.4	238.0	258.7	-74.3	-20.7	0.0
	Uljin	12	176.5	111.6	169.3	176.5	-64.9	-7.2	0.0
	Mokpo	24	405.8	405.8	405.8	405.8	0.0	0.0	0.0
	Jangheung	46	583.4	583.4	583.4	583.4	0.0	0.0	0.0
	Jeju	27	355.8	355.8	355.8	355.8	0.0	0.0	0.0
GLADYS(9112)	Daegwallyeong	2	280.3	280.3	280.3	280.3	0.0	0.0	0.0
	Uljin	12	302.0	302.0	302.0	302.0	0.0	0.0	0.0
	Pohang	16	347.9	347.9	347.9	347.9	0.0	0.0	0.0
	Ulsan	20	461.1	461.1	461.1	461.1	0.0	0.0	0.0
	Busan	22	460.9	460.9	460.9	460.9	0.0	0.0	0.0
NARI(0711)	Jeju	27	421.0	421.0	421.0	421.0	0.0	0.0	0.0
	Seongsan	28	218.5	178.0	200.0	218.5	-40.5	-18.5	0.0
	Goheung	48	239.0	239.0	239.0	239.0	0.0	0.0	0.0
	Namhae	60	91.5	91.5	91.5	91.5	0.0	0.0	0.0

Supplementary Table S3. The number of TCs affecting two study areas among the different radius (500-700km) in the different period (1951-2012 or 1973-2012)

Radius(km)	1951-2012		1973-2012	
	KP	Tokyo	KP	Tokyo
500	306	328	192	197
600	355	402	217	250
700	390	475	241	291

Supplementary Figure

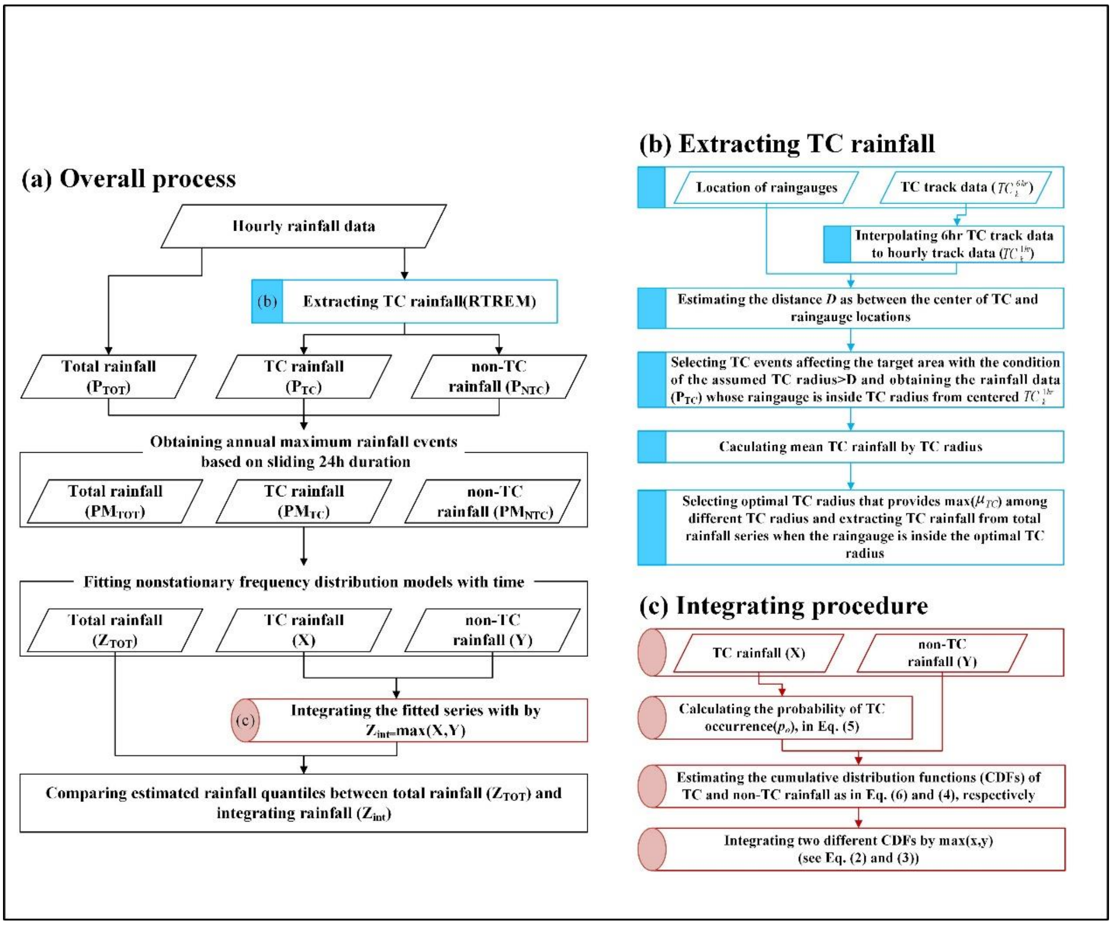

Supplementary Figure S1. (a) Overall process of the proposed approach and (b) procedure of extracting TC rainfall and (c) integrating procedure for TC and non-TC rainfall

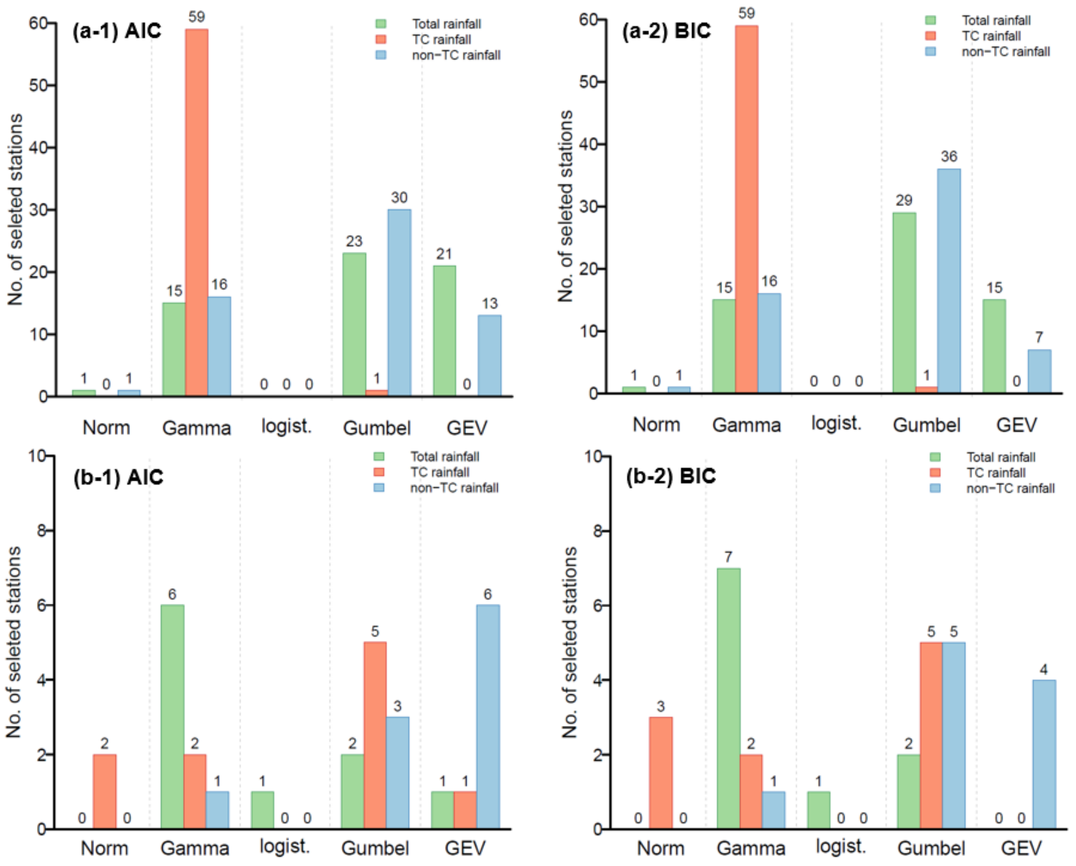

Supplementary Figure S2. Summary of goodness of fit test (AIC and BIC) of five different probability distributions such as normal, Gamma, logistic, Gumbel and GEV distributions for (a) 60 rainfall stations in South Korea and (b) 10 rainfall stations in Tokyo

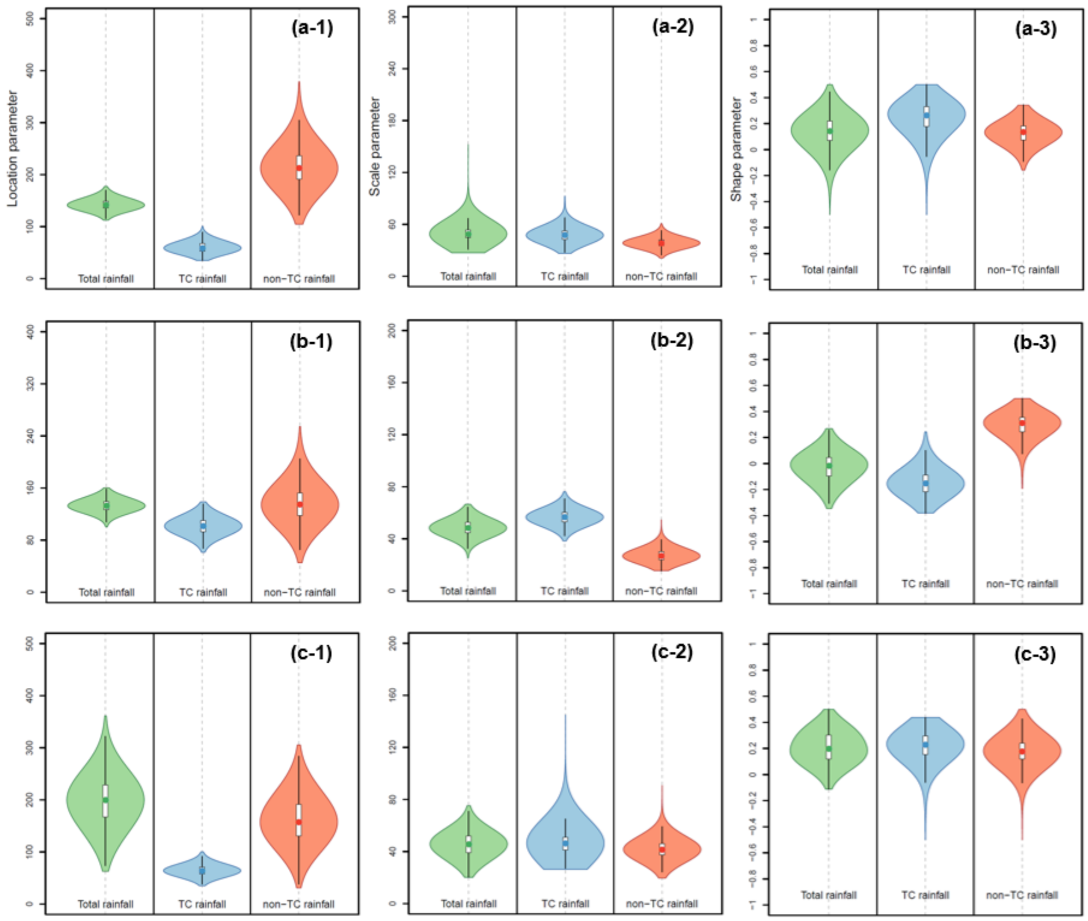

Supplementary Figure S3. Empirical probability distribution for location (left panels), scale (center panels), and shape parameters (right panels) of GEV distribution estimated by bootstrap-based methods (1000set) grouped by upper quartile, median, and lower quartile using violin plot (box plot-density trace synergism) for (a) Busan, (b) Hachioji, (c) Seogwipo
